# A case of canine visceral leishmaniasis of unknown origin in Curitiba (state of Paraná, Brazil) treated successfully with miltefosine

**DOI:** 10.1590/S1984-29612023026

**Published:** 2023-05-15

**Authors:** Gustavo Gonçalves, Monique Paiva Campos, Thais Cristina Tirado, Dayane Domingos Negrão, Gabriela Mayoral Pedroso da Silva, Ana Paula Coninck Mafra Poleto, Tatianna Paula Hartin, Juliana Batista Andrade Silva, Marilia de Melo Santos de Castilhos, Fabiano Borges Figueiredo

**Affiliations:** 1 Laboratório de Biologia Celular, Instituto Carlos Chagas, Fundação Oswaldo Cruz - Fiocruz-PR, Curitiba, PR, Brasil; 2 Laboratório de Referência em Leishmanioses, Instituto Carlos Chagas, Fundação Oswaldo Cruz - Fiocruz-PR, Curitiba, PR, Brasil; 3 Clínica Veterinária Petite Gatô, Curitiba, PR, Brasil; 4 Departamento de Saúde do Estado do Paraná, Divisão de Doenças Transmitidas por Vetores - DVDTV, Curitiba, PR, Brasil; 5 Centro de Controle de Zoonoses, Secretaria de Saúde de Curitiba, Curitiba, PR, Brasil; 6 2° Escritório Regional de Saúde, Departamento de Saúde do Estado do Paraná, Curitiba, PR, Brasil

**Keywords:** Possible autochthonous transmission, Migonemyia migonei, *Brumptomyia* spp., Leishmania infantum, visceral canine leishmaniasis, Possível transmissão autóctone, Migonemyia migonei, *Brumptomyia* spp., Leishmania infantum, leishmaniose visceral canina

## Abstract

There are no records of autochthonous cases of canine visceral leishmaniasis in the city of Curitiba, Paraná state, Brazil. In 2020, a male French bulldog (CW01), approximately 2 years old was taken by its owners to a private veterinarian clinic. The suspicion of CVL was confirmed by means of a serology test (ELISA/IFAT reagent), rapid chromatographic immunoassay (DPP®) (ELISA - Biomanguinhos®), parasitological culture and quantitative polymerase chain reaction (qPCR). The animal routinely frequented parks in Curitiba and was taken on several trips to the municipalities of Bombinhas and Balneário Camboriú (Santa Catarina) and to Matinhos (Paraná) where CVL had not previously been reported. Treatment was initiated orally with Milteforan™ which resulted in a significant reduction in the parasitic load. The suspicion of autochthony was investigated through entomological research. A total of 10 traps were installed, one at the animal’s home, seven in adjacent city blocks and two in a forest edge. No sandflies were trapped in the dog’s home and adjacent houses. The traps in the forest edge caught one *Migonemyia migonei* female and five *Brumptomyia* spp. females. This case serves as a warning of the possible introduction of CVL in the city of Curitiba.

## Resumo

Não há registros de casos autóctones de leishmaniose visceral canina (LVC) no município de Curitiba, Paraná, Brasil. Em 2020, um "bulldog" francês macho (CW01), com aproximadamente 2 anos de idade, foi levado por seus donos a uma clínica veterinária particular. A suspeita de LVC foi confirmada por meio de teste sorológico (reagente ELISA/IFAT), imunoensaio cromatográfico rápido (DPP®) (ELISA - Biomanguinhos®), cultura parasitológica e reação quantitativa em cadeia da polimerase (qPCR). O animal frequentava, rotineiramente, parques de Curitiba e foi levado em diversas viagens para Bombinhas e Balneário Camboriú (Santa Catarina) e para Matinhos (Paraná), onde a LVC não havia sido relatada anteriormente. O tratamento foi iniciado por via oral com Milteforan™ (Virbac) o que resultou em redução significativa da carga parasitária. A suspeita de autóctone foi investigada por meio de pesquisa entomológica. Foram instaladas 10 armadilhas, uma na casa do animal, sete em quarteirões adjacentes e duas na borda da mata. Nenhum inseto foi capturado na casa do proprietário do animal e nas casas adjacentes. As armadilhas na borda da mata capturaram uma fêmea de *Migonemyia migonei* e cinco *Brumptomyia* spp. fêmeas. Este caso serve de alerta para a possível introdução da LVC na cidade de Curitiba.

Leishmaniasis is a parasitic disease caused by protozoan parasites of the genus *Leishmania* and transmitted by the bite of infected sandflies. The most severe form, visceral leishmaniasis (VL), has clinical features of severe evolution in humans (WHO, 2022), aggravated by the occurrence of a zoonotic cycle in South America and in the Mediterranean region (Werneck, 2014). In this context, *Leishmania infantum* (syn = *Leishmania chagasi*) is the most important etiological agent involved, and domestic dogs (*Canis familiaris*) are the main reservoir in urban environments (Brasil, 2014). Canine enzootic disease precedes the occurrence of human cases (Brasil, 2014), and control of the zoonotic cycle is still the main challenge in disease control today (Werneck, 2014).

An average of 3,500 new cases are recorded annually in Brazil, resulting in the death of approximately 250 humans. At first, the disease occurred mostly in small towns and rural areas in Brazil’s northeast region. However, in recent years, it has expanded rapidly to more populated urban areas, and an increasing number of cases have been reported in the north, center-west, southeast and even south of the country, which was free of the disease up to the 2000s (Brasil, 2022).

Alternatives for treatment of the disease are limited and unsatisfactory. For over 60 years, human treatment involved the use of pentavalent antimonials (Chakravarty & Sundar, 2010). Subsequently, new drugs were developed that still make up a small arsenal, which characterizes the disease as neglected. Current treatments are applied using three main drugs: pentavalent antimonials (drug of first choice), amphotericin B and miltefosine (Hefnawy et al., 2017).

The treatment of canine visceral leishmaniasis with miltefosine started in Brazil in 2016 (Brasil, 2016). However, failures have been reported in this treatment using solely miltefosine and in association with other drugs, in which the decrease in canine symptoms was not accompanied by parasite clearance (Andrade et al., 2011). Thus, treatment is still not considered an effective measure because, in addition to the risk of parasite resistance (Gonçalves et al., 2021), relapses are frequent and dogs may be a source of infection by the invertebrate host even weeks after the end of treatment, despite clinical cure (Chappuis et al., 2007).

There are no records of autochthonous cases of canine visceral leishmaniasis in the city of Curitiba. However, studies such as those of Thomaz-Soccol et al. (2009) and Frehse et al. (2010) observed positivity in dogs in this city, some of them allochthonous cases and some asymptomatic. These cases underscore the importance of epidemiological surveillance studies in the city, since domestic dogs are the main reservoir of the disease in urban environments, and once the vector is present, the transmission cycle can be established in new areas through the arrival of infected animals (Maia-Elkhoury et al., 2008). This article reports a case of canine visceral leishmaniasis of unknown origin, treated successfully with miltefosine, in Curitiba, state of Paraná, Brazil.

In January 2020, a male French bulldog (CW01), approximately 2 years old, non-neutered, exhibiting alopecia, skin desquamation and crusting, dermatitis, and lymphadenomegaly, was taken by its owners to a private veterinarian clinic. The staff veterinarian suspected canine visceral leishmaniasis (CVL) and ordered a blood count and serological tests.

After clinical and laboratory evaluation, it was found that the animal had normocytic and normochromic anemia, hyperproteinemia, but no biochemical alterations in liver and kidney functions. The suspicion of CVL was confirmed by means of a serology test (ELISA/IFAT reagent).

Following the recommendations of the Ministério da Saúde to confirm CVL, a new blood sample was collected and sent to the municipality’s official laboratory (LACEN-PR), where infection by *L. infantum* was confirmed in a rapid chromatographic immunoassay (DPP®) (ELISA - Biomanguinhos®).

As this case occurred in an area with no reports of transmission, other confirmatory tests such as parasitological culture and quantitative polymerase chain reaction (qPCR) were suggested and performed by the reference laboratory in Leishmaniasis (ICC-Fiocruz PR). For qPCR, intact skin fragments were collected using a 3 mm diameter punch, placed in a sterile flask free of RNase and DNase, and stored at -20 °C. For the parasitological culture, another fragment of skin, bone marrow and lymph node aspirates were collected. Samples were kept at 4 °C for 24 hours and then seeded in Novy-MacNeal-Nicole (NNN) medium and Schneider medium (supplemented with 10% SBF) and examined weekly for one month under an optical microscope to look for forms of promastigotes of the parasite. Infection was confirmed and the parasite was characterized as *L. infantum* using qPCR with species-specific primers. After DNA extraction, the sample was amplified using the TaqMan® system on the StepOne™ platform (Applied Biosystems®). The TaqMan® MGB probe (FAM-5’AAAAAAATGGGTGCAGAAAT-3’-NFQM-3GB) and PCR with primers LEISH-1 (5’-AACTTTCTGGCTCCGGGTAG-3’) and LEISH-2 (5’-ACCCCCAGTT TCCCGCC-3’) were designed to target conserved regions of *L. infantum* kDNA (Francino et al., 2006). Samples were amplified on the StepOne™ platform (Applied Biosystems®). According to the stages suggested by Leishvet (Solano-Gallego et al., 2011), this dog was in stage II (moderate disease), with a prognosis of good to moderate.

In describing the dog’s medical history, its owners stated that the animal routinely frequented parks in Curitiba and was taken on several trips to the municipalities of Bombinhas and Balneário Camboriú in the state of Santa Catarina and to Matinhos, a coastal town in the state of Paraná, where CVL had not previously been reported according to official data (Brasil, 2022). The dog had never had a blood transfusion, and tests on the animal’s dam and sire could not be carried out.

The suspicion of CVL was confirmed by test results (T0, March 2020). A qPCR of the dog’s skin revealed a parasite load of ≤ 492 gEq, while DNA sequencing (Sanger method) confirmed that the growth of promastigote forms in parasite culture of skin and bone marrow was *Leishmania infantum* (MCAN/BR/19/CW01), with 100% homology.

Treatment was initiated orally with Milteforan™ (Virbac), following the manufacturer’s instructions of 2 mg/kg of body weight, once a day, for 28 consecutive days, as well as domperidone 0.5 mg/kg every 24 hours, orally, for 30 days. In addition, 10mg/kg of allopurinol was applied orally every 12 hours.

To control the parasite load and monitor for adverse effects, new tests were performed after 4 months of treatment (T1, July 2020). The qPCR revealed a significant reduction in the parasite load (0.1 gEq) of the animal, which was asymptomatic and showed no significant adverse effects. For follow-up, these tests were performed 1 year and 3 months after treatment (T2, October 2021) and 2 years after treatment (T3, August 2022), showing a low parasite load remained (0.1 gEq), without clinical signs. Upon the diagnosis of CVL, the lifelong use of an insect repellent dog collar was recommended to protect against sandflies.

The suspicion of autochthony was investigated through entomological research by the Paraná State Health Department (SESA) and the Curitiba Municipal Health Department (Surveillance and Zoonoses Unit), using CDC and Shannon light traps in the animal’s home and its surroundings for three consecutive days. A total of 10 traps were installed, one at the animal’s home, seven in adjacent city blocks and two in a forest edge located approximately 2.9 km from the home.

No sandflies were trapped in the dog’s home and adjacent houses. The traps in the forest edge caught one *Migonemyia migonei* female and five *Brumptomyia* spp. females, although the latter could not be identified to a specific level due to the similarity between the females that make up the genus.

This case serves as a warning of the possible introduction of CVL in the city of Curitiba. However, since the probable site of infection (PSI) of the case is unknown, the city is still considered to be free of transmission of this disease.

The patient in this report was taken to the municipality of Bombinhas (Santa Catarina, Brazil) on several occasions, and although there is no evidence of transmission of the disease in the aforementioned municipality, transmission is known to occur in other nearby locations, such as the capital Florianópolis (Steindel et al., 2013). Moreover, the dog was purchased from a kennel in the metropolitan region of Curitiba, but this information did not allow for identification of the kennel and the animal’s dam and sire, thus preventing the performance of tests. This is a troubling situation, because if the patient’s dam and/or sire are infected with the parasite, they can produce several generations of litters that are also positive, contributing to the spread of infected animals in an area initially not endemic for the disease.

Curitiba and its metropolitan area are located about 100 km from the region of Vale do Ribeira, which is widely known for the presence of sandflies. In this paper, we report for the first time the presence of *Mi. migonei*, mainly known for the transmission of cutaneous leishmaniasis (CL), in Curitiba, the capital of the state of Parana, which may be related to the registration of CL cases in and around Curitiba from 2001 to 2019 (Almeida et al., 2022). However, this species has also previously been associated with the transmission of visceral leishmaniasis (Moya et al., 2015), making it of medical and veterinary importance in *Leishmania* spp. transmission. Additionally, *Brumptomyia s*pp. have been reported to transmit *L. braziliensis* in northern Brazil (Araujo-Pereira et al., 2020), although previous studies described these species as non-anthropophilic (Rêgo et al., 2019). This genus has also been reported in the states of São Paulo (Cutolo et al., 2013) and Paraná (Bianchi Galati et al., 2007), but has not been associated with the transmission of leishmaniasis. Published data report the presence of *Lutzomyia neivai*, *Lu. fischeri* and *Lu. ayrozai*, which are vectors of cutaneous leishmaniasis (CL), along the coast of Santa Catarina (Marcondes et al., 2005). Species of the genus *Lutzomyia* have already been reported as naturally infected with *L. infantum* in endemic areas for visceral leishmaniasis, with indications of a possible participation of these species in disease transmission (Savani et al., 2009; Falcão de Oliveira et al., 2017), alerting to the possible role of sand fly species not traditionally involved in the transmission of the parasite in the anthropozoonotic cycle of the disease.

Another interesting aspect of this case is the success of the treatment, which can be attributed to the fact that this was a young dog without comorbidity, with early diagnosis and correct treatment, which allowed rapid and continuous improvement of the patient’s clinical condition. It is worth noting that there is no parasitological cure for CVL, only clinical control; hence, a repellent collar must be worn throughout the animal’s lifetime and must be monitored continuously.

Since 2016, health authorities of the Zoonotic Disease Surveillance Unit and the Paraná State Health Department have carried out periodic CVL monitoring of dogs in the municipality. In addition, entomological surveys were carried out in 2018 and 2020, and over the years, awareness among veterinarians has been raised by an increase in notifications, with all cases investigated and followed up.

Canine visceral leishmaniasis is a complex disease that requires integrated actions for its control in animals and humans. In this report, we demonstrate the importance of interaction and communication among public agencies (LACEN - PR, Fiocruz PR, SESA, UVZ) and independent veterinarians for the diagnosis, treatment and control of this zoonotic disease.
